# Effects of smoking habit change on all-cause mortality and cardiovascular diseases among patients with newly diagnosed diabetes in Korea

**DOI:** 10.1038/s41598-018-23729-0

**Published:** 2018-03-28

**Authors:** Mi Hee Cho, Kiheon Lee, Sang Min Park, Jooyoung Chang, Seulggie Choi, Kyuwoong Kim, Hye-Yeon Koo, Ji-Hye Jun, Sung Min Kim

**Affiliations:** 1 Department of Family Medicine, Seoul National University Hospital, Seoul, 03080 Republic of Korea; 2 Department of Family Medicine, Seoul National University Bundang Hospital, Seongnam-si, Gyeonggi-do, 13620 Republic of Korea; 3 Department of Family Medicine, College of Medicine, Seoul National University, Seoul, 03080 Republic of Korea; 4 Department of Biomedical Sciences, Seoul National University Graduate School, Seoul, 03080 Republic of Korea

## Abstract

This study aimed to investigate the effects of smoking habit change on the risks of all-cause mortality and cardiovascular diseases (CVDs) among patients with newly diagnosed diabetes using the Korean National Sample Cohort data. Survival regression analyses for the risks of all-cause mortality and CVDs were performed. Quitters without body mass index (BMI) change (adjusted hazard ratio [aHR], 0.68; 95% confidence interval [CI], 0.46–1.00) and quitters with BMI loss (aHR, 1.76; 95% CI, 1.13–2.73) showed significantly reduced and substantially the increased risk of all-cause mortality, respectively, compared with sustained smokers. Smoking reduction after diabetes diagnosis may have potential positive effects. However, definite benefits on the health outcomes were not identified in this study. Participants who started smoking after diabetes diagnosis had higher risks of all-cause mortality and CVDs than those who were never smokers or ex-smokers, although not statistically significant. In conclusion, smoking cessation after diabetes diagnosis could reduce the risks of all-cause mortality and cardiovascular events among patients with newly diagnosed diabetes when accompanied by proper weight management. Therefore, physicians should advice patients with newly diagnosed type 2 diabetes on the importance of smoking cessation in combination with long-term weight management to maximize the benefits of smoking cessation.

## Introduction

Cigarette smoking is a well-known risk factor for several types of cancer and cardiovascular diseases (CVDs), including cerebrovascular disease and ischemic heart disease (IHD), in general population^1–4^. Several studies have proven that smoking cessation has considerable health benefits, especially in the reduction of the risks of CVDs^2,4^. Based on the health report of the Organization for Economic Cooperation and Development (OECD), the rate of mortality caused by IHD and cerebrovascular diseases has been gradually reduced in most Western countries, which is reported to be associated with the increased rate of smoking cessation^5^. However, the rate of deaths related to CVDs has been increasing in Korea, although this country has relatively lower incidence rates of CVDs than western OECD countries^5,6^.

Patients with diabetes are generally considered as a high risk group of CVDs with higher mortality rate than those without diabetes^7,8^. Smoking has been reported as not only one of the risk factors for diabetes in general population^9–11^ but also one of factors strongly associated with mortality^7,12^ and diabetic complications among patients with type 2 diabetes^13,14^. Thus, the clinical guidelines for diabetes have recommended smoking cessation as a modifiable factor in the reduction of health risks among patients with diabetes^15–17^. However, although people who are newly diagnosed with chronic diseases, such as diabetes and hypertension, are expected to adopt healthy behaviors, such as smoking cessation and decreased alcohol consumption^18^, the smoking rate among patients with diabetes do not appear to be on decline^19^.

The prevalence rate of diabetes among adults aged ≥ 30 years old in Korea is approximately 13%, based on the recent report of the Korean Diabetes Association, and this rate is higher than the average rates in OECD countries^20^. Furthermore, the smoking rates in Korea are also among the highest in the world, which are approximately 23% among Koreans aged ≥ 15 years old and > 30% among Korean male adults^5^. To the very best of our knowledge, research on the effects of smoking habit change after diabetes diagnosis on health outcomes has not been systemically investigated, despite the high prevalence rates of diabetes and smoking, especially in Asian countries. Thus, in this large retrospective cohort study, we investigated the patterns of smoking habit change after diabetes diagnosis. Additionally, we determined the effects of smoking habit change on health outcomes, such as the risks of all-cause mortality and CVDs, of patients with newly diagnosed diabetes in Korea.

## Results

**Figure 1:**
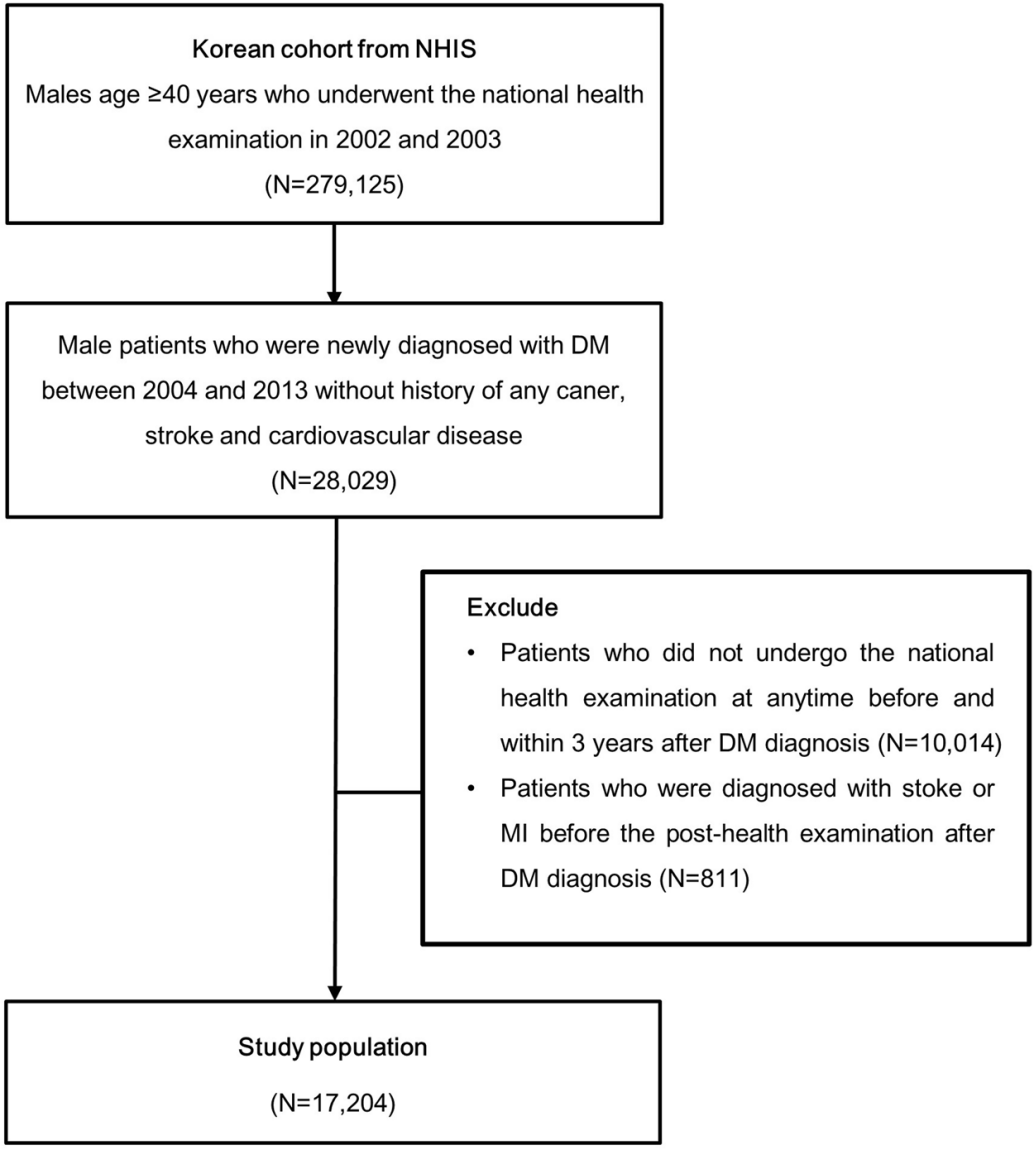
Study population. The study population includes patients who underwent health examination within 3 years before and after diabetes diagnosis. NHIS, National Health Insurance Service; N, number of patients; DM, diabetes mellitus; MI, myocardial infarction.

A total of 28,029 male patients were newly diagnosed with type 2 diabetes between 2004 and 2013. Considering that the status of smoking habit change was the main independent variable in this study, patients who did not undergo the health examination conducted by the National Health Insurance Service (NHIS) within 3 years before and after diabetes diagnosis (N = 10,014) were excluded. Furthermore, patients who were diagnosed with stroke or myocardial infarction (MI) before undergoing health checkup after diabetes diagnosis (N = 811) were eliminated from the study. A total of 17,204 male patients were finally included in the study population (Figure 1). Table 1 presents the basal demographic and clinical characteristics of patients with newly diagnosed type 2 diabetes based on the status of smoking habit change. The median age, body mass index (BMI), and fasting glucose level of all patients were 52 years, 24.84 kg/m^2^, and 104 mg/dL, respectively (Table 1). The smoking rates before and after diabetes diagnosis were 33.0% and 29.4%, respectively. An interesting finding was that approximately 5% of patients with newly diagnosed diabetes started or relapsed smoking even after the diagnosis.

**Table 1:** Basal demographic and clinical characteristics of study population.

	All		Non-reducer		Reducer		Quitter		Never smoker		Ex-smoker		Starter	
	N	%	N	%	N	%	N	%	N	%	N	%	N	%
Patient No.	17,204	100	3,599	20.92	544	3.16	1,538	8.94	5,341	31.05	5,274	30.66	908	5.28
Age, years														
Median (IQR)	52.0	(14.0)	49.0	(11.0)	50.5	(13.0)	51.0	(13.0)	55.0	(14.0)	52.0	(14.0)	51.0	(13.0)
40–49	6,787	39.45	1,835	50.99	247	45.40	638	41.55	1,610	30.14	2,053	38.93	403	44.38
50–59	5,987	34.80	1,201	33.37	183	33.64	547	35.57	1,915	35.85	1,832	34.74	309	34.03
60–69	3,709	21.56	494	13.73	102	18.75	300	19.51	1,488	27.86	1,152	21.84	173	19.05
≥70	721	4.19	69	1.92	12	2.21	52	3.38	328	6.14	237	4.49	23	2.53
Income status														
Quartile 1	5,506	32.00	1,280	35.57	197	36.21	533	34.66	1,682	31.49	1,537	29.14	277	30.51
Quartile 2	3,342	19.43	742	20.62	113	20.77	285	18.53	1,026	19.21	959	18.18	217	23.90
Quartile 3	4,879	28.36	1,011	28.09	151	27.76	420	27.31	1,473	27.58	1,572	29.81	252	27.75
Quartile 4	3,488	20.21	566	15.73	83	15.26	300	19.51	1,160	21.72	1,206	22.87	162	17.84
CCI														
0	13,367	77.70	2,785	77.38	412	75.74	1,166	75.81	4,099	76.75	4,205	79.73	700	77.09
1	3,068	17.83	662	18.39	102	18.75	302	19.64	973	18.22	868	16.46	161	17.73
≥2	769	4.47	152	4.22	30	5.51	70	4.55	269	5.04	201	3.81	47	5.18
Alcohol consumption														
<3 time/month	9,464	55.01	1,476	41.01	236	43.38	694	45.12	3,549	66.45	2,898	54.95	611	67.29
1–2 time/week	3,933	22.86	1,061	29.48	134	24.63	454	29.52	970	18.16	1,176	22.30	138	15.20
≥3 time/week	3,202	18.61	886	24.62	152	27.94	352	22.89	699	13.09	982	18.62	131	14.43
Physical activity														
None	6,053	35.18	1,360	37.79	226	41.54	562	36.54	2,004	37.52	1,456	27.61	445	49.01
1–2 time/week	4,149	24.12	995	27.65	125	22.98	444	28.87	1,242	23.25	1,167	22.13	176	19.38
>3 time/week	6,877	39.97	1,237	34.37	192	35.29	528	34.33	2,043	38.25	2,614	49.56	263	28.96
BMI (kg/m^2^)														
Median (IQR)	24.84	(3.62)	24.74	(3.67)	24.74	(4.13)	24.68	(3.56)	24.90	(3.56)	24.91	(3.47)	24.74	(3.72)
<18.5	199	1.16	57	2.76	15	2.76	28	1.82	37	0.69	50	0.95	12	1.32
18.5–22.9	4,138	24.05	978	29.60	161	29.60	381	24.77	1,281	23.98	1,124	21.31	213	23.46
23.0–24.9	4,974	28.91	1,012	24.26	132	24.26	438	28.48	1,589	29.75	257	29.31	257	28.30
≥25.0	7,890	45.86	1,552	43.20	235	43.20	691	44.93	2,434	45.57	426	48.39	426	46.92
BMI change (kg/m^2^)^a^														
Median (IQR)	−0.06	(1.45)	−0.08	(1.52)	−0.16	(1.59)	0.08	(1.66)	−0.09	(1.40)	−0.07	(1.36)	−0.25	(1.53)
Blood pressure (mmHg)														
Median (IQR)^b^	130	(20)	130	(20)	130	(20)	130	(20)	130	(21)	130	(20)	130	(20)
<120/80	2,943	17.11	706	19.62	97	17.83	307	19.96	825	16.04	846	16.04	162	17.84
120/80–139/89	8,454	49.14	1,749	48.60	253	46.51	731	47.53	2,551	51.73	2,728	51.73	442	48.68
140/90–159/89	4,012	23.32	786	21.84	124	22.79	349	22.69	1,330	22.92	1,209	22.92	214	23.57
≥160/90	1,792	10.42	357	9.91	70	12.87	151	9.82	634	9.29	490	9.29	90	9.91
Total cholesterol (mg/dL)														
Median (IQR)	202	(50)	203	(51)	205	(51)	202	(49)	199	(49)	204	(50)	202.0	(51)
<200	8,925	51.88	1,755	48.76	275	50.55	504	51.50	2,971	55.63	2,675	50.72	457	50.33
200–239	5,639	32.78	1,231	34.20	173	31.80	504	32.77	1,675	31.36	1,761	33.39	295	32.49
≥240	2,625	15.26	609	16.92	95	17.46	240	15.60	688	12.88	837	15.87	156	17.18
Fasting glucose (mg/dL)														
Median (IQR)	104	(34)	106	(39)	108	(43)	104	(34)	105	(35)	102	(31)	104	(32)
<100	4,642	26.98	889	24.70	139	25.55	464	30.17	1,514	28.35	1,375	26.07	261	28.74
100–125	5,741	33.37	1,076	29.90	173	31.80	494	32.12	1,820	34.08	1,903	36.08	275	30.29
≥126	6,814	39.61	1,631	45.32	231	42.46	580	37.71	2,004	37.52	1,996	37.85	372	40.97

N, number of patients; CCI, Charlson comorbidity index; BMI, body mass index; IQR, interquartile range.

^a^BMI change was calculated by subtracting the BMI measured at the health examination before diabetes diagnosis from that measure at the health examination after diabetes diagnosis.

^b^Median blood pressure was presented using the systolic blood pressure measured at the health examination before diabetes diagnosis.

Table 2 demonstrates the crude incidences and hazard ratios of all-cause mortality for each group. Based on the results of the survivor analysis, quitters without BMI change had significantly reduced the risk of all-cause mortality in both age-adjusted (adjusted hazard ratio [aHR], 0.63; 95% confidential interval [CI], 0.43–0.92) and multivariate-adjusted (aHR, 0.68; 95% CI, 0.46–1.00) models compared with sustained smokers (Table 2). Similarly, quitters with BMI gain (aHR, 0.67; 95% CI, 0.36–1.24) also showed decreased risk of death, although not statistically significant. However, quitters with BMI loss (aHR, 1.76; 95% CI 1.13–2.73) showed substantially increased risk of mortality (Table 2).

**Table 2:** Incidence rates and hazard ratios of all-cause mortality based on smoking habit change among patients with newly diagnosed diabetes who were smokers before diabetes diagnosis.

	N	Person-year	Crude incidence rate (per 10^5^person-year)		Age adjusted HR			Multivariate adjusted HR ^a^		
			IR	95% CI	aHR	P	95% CI	aHR	P	95% CI
All-cause mortality										
All	248	10,439,049	2.38	2.10–2.69	—	—	—	—	—	—
Non-reducer	147	6,499,490	2.26	1.92–2.66	1.00	—	—	1.00	—	—
Reducer	34	1,024,191	3.32	2.37–4.65	1.23	0.27	0.85–1.79	1.22	0.31	0.83–1.79
Quitter with BMI loss	24	539,588	4.45	2.98–6.64	1.58	0.04	1.03–2.44	1.76	0.01	1.13–2.73
Quitter without BMI change	32	1,690,769	1.89	1.34–2.68	0.63	0.02	0.43–0.92	0.68	0.05	0.46–1.00
Quitter with BMI gain	11	685,011	1.61	0.89–2.90	0.64	0.16	0.35–1.19	0.67	0.21	0.36–1.24

N, number of events; IR, incidence rate; HR, hazard ratio; aHR, adjusted hazard ratio; CI, confidence interval; BMI, body mass index.

^a^Multivariate-adjusted model was adjusted for age, income status, Charlson comorbidity index score, alcohol consumption, physical activity, smoking status, BMI, blood pressure, and fasting serum glucose and cholesterol levels.

The results of risk assessment for CVDs for each of smoking habit change were presented in Table 3. Among the quitters, patients with BMI loss showed increased risk of CVDs (aHR, 1.09; 95% CI, 0.71–1.68), whereas those without BMI change (aHR, 0.79; 95% CI, 0.58–1.07) or with BMI gain (aHR, 0.94; 95% CI, 0.60–1.47) displayed decreased risk of CVDs, although not statistically significant (Table 3). The hazard ratio of CVDs in patients with smoking reduction (aHR, 0.75; 95% CI, 0.51–1.10) decreased, while risk of mortality in those increased (aHR, 1.22; 95% CI, 0.83–1.79), although without statistical significance (Table 3). Table 4 presents the risks of mortality and CVDs among smoking starters after diabetes diagnosis. The hazard ratios of all-cause mortality and CVDs in the starter group were 1.00 (95% CI, 0.67–1.49) and 1.28 (95% CI, 0.94–1.75), respectively, compared those of the never smoker or ex-smoker group (Table 4).

**Table 3:** Incidence rates and hazard ratios of cardiovascular diseases based on smoking habit change among patients with newly diagnosed diabetes who were smokers before diabetes diagnosis.

	N	Person-year	Crude incidence rate (per 10^5^person-year)		Age adjusted HR			Multivariate adjusted HR ^a^		
			IR	95% CI	SHR	P	95% CI	aHR	P	95% CI
Cardiovascular diseases										
All	349	10,162,561	3.43	3.09–3.81	—	—	—	—	—	—
Non-reducer	219	6,326,089	3.46	3.03–3.95	1.00	—	—	1.00	—	—
Reducer	29	1,001,927	2.89	2.01–4.17	0.73	0.14	0.50–1.07	0.75	0.14	0.51–1.10
Quitter with BMI loss	23	525,587	4.38	2.91–6.59	1.07	0.23	0.70–1.63	1.09	0.70	0.71–1.68
Quitter without BMI change	56	1,640,199	3.41	2.63–4.44	0.83	0.12	0.62–1.11	0.79	0.13	0.58–1.07
Quitter with BMI gain	22	668,759	3.29	2.17–5.00	0.90	0.20	0.58–1.40	0.94	0.79	0.60–1.47

N, number of events; IR, incidence rate; HR, hazard ratio; aHR, adjusted hazard ratio; CI, confidence interval; BMI, body mass index.

^a^Multivariate-adjusted model was adjusted for age, income status, Charlson comorbidity index score, alcohol consumption, physical activity, smoking status, BMI, blood pressure, and fasting serum glucose and cholesterol levels.

**Table 4:** Multivariate-adjusted hazard ratios with 95% confidence intervals of all-cause mortality and cardiovascular disease based on smoking habit change among patients who were nonsmokers before diabetes diagnosis.

	All Patients		All-cause mortality				Cardiovascular diseases			
	N	%	N	aHR^a^	95% CI	P for trend	N	aHR^a^	95% CI	P for trend
Never smoker	5,341	46.35	235	1.00	—	0.77	303	1.00	—	0.16
Ex-smoker	5,274	45.77	162	1.05	0.86–1.29		216	1.06	0.88–1.27	
Starter	908	7.88	32	1.00	0.67–1.49		52	1.28	0.94–1.75	

N, number of patients; aHR, adjusted hazard ratio; CI, confidence interval.

^a^Multivariate-adjusted model was adjusted for age, income status, Charlson comorbidity index score, alcohol consumption, physical activity, smoking status, BMI, blood pressure, and fasting serum glucose and cholesterol levels.

## Discussion

In this national cohort study, the risk of all-cause mortality among quitters without BMI change was significantly reduced compared with sustained smokers in the group of patients with newly diagnosed diabetes. Additionally, the benefits of smoking cessation on the risks of all-cause mortality and CVDs were attenuated by BMI loss or gain after quitting smoking. To the very best of our knowledge, this study is the first to evaluate the effects of smoking habit changes, along with BMI changes after smoking cessation, on the risks of mortality and cardiovascular events among male patients with newly diagnosed diabetes in Asia.

When patients are newly diagnosed with diabetes, they are generally expected to engage in a healthy lifestyle, such as starting physical activities and smoking cessation. However, this study found that the smoking rate only dropped from 33% to 29% after diabetes diagnosis and that 5% of patients who never or used to smoke began or restarted smoking even after diabetes diagnosis. Therefore, such high smoking rate even after the diagnosis suggests that increased efforts should be placed in educating patients on the importance of smoking cessation.

The risk of death was found to be substantially decreased among patients who quit smoking after diabetes diagnosis, unless BMI difference after smoking cessation was not >1.0 kg/m^2^. The hazard ratios of mortality and CVD events for the quitter group with BMI gain also decreased, although without statistical significance. However, the quitters with BMI gain had higher risks than quitters without BMI change. This finding could be attributed to the fact that the effect of CVD risk reduction due to smoking cessation might be attenuated by weight gain, which is consistent with the results of previous studies^21,22^. The beneficial effects of smoking cessation outweigh the risks associated with weight gain after quitting smoking^21,22^. However, weight gain has been recognized as a sole risk factor of CVDs^23,24^. Furthermore, weight gain can occur for more than 20 years after smoking cessation and finally offset the benefits of quitting smoking^22^. Thus, physicians should provide patients with long-term weight management interventions in combination with smoking cessation.

In contrast, quitters with BMI loss showed significant increase in the risk of mortality, which was even higher than sustained smokers. Additionally, those patients also had increased risk of cardiovascular events, although not statistically significant. Based on these findings, quitters with BMI loss after smoking cessation may have experienced increased weight loss caused by either poorly controlled diabetes or presence of more serious disease, such as cancer, leading to increased mortality. Additionally, according to the results of age-stratified analysis in this study, the change in the risks of death and CVDs in the group of quitters with BMI loss was more noticeable among elderly patients aged ≥ 60 years old than their younger counterparts (Supplementary Table S1). These findings were consistent with those of previous researches, which reported that weight loss was associated with increased mortality even among the elderly without severe diseases, suggesting that weight loss could be an independent risk factor of increased mortality among the elderly^25,26^. Previous studies have proposed several possible mechanisms of increased health risks due to weight loss among the elderly^26–28^. One of these mechanisms is that elderly people who lose weight mainly experience loss of muscle mass, which results in sarcopenic obesity, thus increasing the risk of CVDs due to increased insulin resistance, hypertension, and dyslipidemia^28^. This mechanism could be one of the possibilities explaining the increased health risks among patients with diabetes who lost weight after smoking cessation. However, considering the nature of the administrative database used in this study, this proposed explanation could not be proven. Hence, further study would be needed to determine the reasons for the decreased BMI among patients who quit smoking after diagnosis and the increased health risks among these patients.

Smoking reduction has been investigated as one of the harm reduction strategies. Although the efficacy of smoking reduction has been a topic of debate, several studies have shown the benefits of this strategy on health outcomes^29,30^. However, reduction in the smoking amount per day did not reduce the mortality rate of patients with diabetes in this study, while smoking reduction tended to decrease the risk of CVDs without statistical significance. This result is consistent with the previous findings^31^ and may suggest the possible benefit of smoking reduction. However, physicians should advise quitting – not just reducing – smoking to patients who are asking whether smoking reduction may be beneficial because the results have been controversial so far. In addition, further research with longer follow-up period would be necessary to better investigate the effects of smoking reduction on health outcomes.

In the subgroup analysis, the risks of mortality and CVDs of smoking starters after diabetes diagnosis, compared with those of never smokers or ex-smokers, were investigated. Smoking starters appeared to be the risky group for CVDs and may even have higher risks than ex-smokers, although without statistical significance. Patients who initiated or relapsed smoking even after diabetes diagnosis may be speculated to have less interest in improving their health, resulting in poor diabetes control or other unhealthy behaviors, which could explain the increased health risks observed among smoking starters. Possible reason for the absence of statistically significant increase in health risks of the smoking starters is the length of the follow-up duration in this study, which might not be long enough to show the cumulative harmful effects of smoking. Research on the health outcomes among smoking starters after a diagnosis of chronic diseases remain to be conducted so far. Therefore, further studies with longer follow-up period are necessary to support these findings and provide a strong recommendation to avoid smoking initiation.

Our study has several strengths. First, it has a large cohort and utilized the national health examination data and claim records. Second, this study took into account the BMI changes after smoking cessation to assess the health risks of patients with diabetes. Third, the number of patients who initiated or relapsed smoking even after diabetes diagnosis was determined. However, despite the strengths and novelty of this study, it has several limitations that need to be considered when interpreting the results. First, smoking habit changes were assessed only based on the patient’s self-reporting without biochemical verification of smoking status, which may result in overestimating the rate of smoking cessation. To chemically verify the smoking status of the participants, a future study that is designed to include biomarker tests, such as urine cotinine levels, is needed. Second, given the nature of the database used in this study, the data set lacked clinical details, such as the causes of the BMI changes and smoking cessation duration in the quitter groups. The BMI loss after smoking cessation could be caused by either intentional or unintentional weight loss due to the presence of other diseases. Therefore, the health outcomes of the participants could vary substantially, depending on the cause of weight loss, which could be one of the confounding factors in the assessment of health risks. Furthermore, smoking cessation duration could also be a factor that needs to be considered in the risk assessment. However, these possible confounding factors were not evaluated in this study. Third, this study was conducted only among male patients, given the considerable difference in the smoking rates between men and women (approximately 40% and 2%, respectively) in the general population of Korea^5^. In previous studies, smoking substantially increased the risk of coronary heart disease among women with type 2 diabetes, and smoking cessation was found to decrease this risk^32,33^. Furthermore, female smoking rate in Korea has increased over the last ten years unlike that in most OECD countries^5^. Thus, a study on the effects of smoking habit changes on the health outcomes of female patients with diabetes should be conducted in the future. Fourth, some biases in terms of the definition of the study population might exist. In this study, the study population only included patients who voluntarily participated in the national health screening examination. Therefore, the health conditions of these patients might be given more attention than those of the general population, resulting in better health outcomes due to the selection bias.

In conclusion, smoking cessation after diabetes diagnosis could be beneficial in the reduction of the risks of all-cause mortality and cardiovascular events among patients with newly diagnosed diabetes. However, the results of our study also showed that the beneficial effects of smoking cessation were attenuated by weight gain. Moreover, weight loss after smoking cessation even increased the risks of all-cause mortality and CVDs. Reduction in the smoking amount per day after diabetes diagnosis may have potential positive effects. However, its definite benefits on the health outcomes were not identified in this study. To that end, our findings have important clinical implications, specifically suggesting that physicians should advice patients with newly diagnosed type 2 diabetes on the importance of smoking cessation in combination with long-term weight management to maximize the benefits.

## Methods

### Data sources and study population

This retrospective cohort study was conducted using the Korean National Health Insurance Service – National Health Screening Cohort (NHIS-HealS, 2002–2013). In Korea, the NHIS provides mandatory universal health insurance to nearly all Koreans (96.9%), and the beneficiaries of the NHIS who were ≥ 40 years old are required to undergo the health checkups biennially^34^. The number of participants in the NHIS-HealS database is approximately 510,000, 10% of randomly selected Koreans who were aged between 40 and 79 years old as of December 2002 and were eligible for the biennial health examination in 2002 and 2003^35^. The registered patients were followed up from January 1, 2002, to December 31, 2013. The study participants were limited to men. Among the eligible patients, 279,125 were males. The results of the national health examination and all medical and demographic information were obtained from the NHIS-HealS database^35,36^. The medical information include the following: use of medical facilities, such as clinic visits as outpatients and hospitals; diagnostic codes based on the codes established by the International Classification of Disease (ICD); and records of prescribed medicines, including dates of prescription, amount dispensed, and number of defined daily doses. The demographic information include the following: age, sex, insurance premium (proxy for income level), disability status, and place of residence.

Newly diagnosed patients were defined based on the International Classification of Diseases, 10^th^ Revision (ICD-10) codes. Among the participants without claimed records of the ICD-10 codes related to type 2 diabetes (E11, E12, and E14) for first the 2 years (in 2002 and 2003), those who visited the hospitals with diabetic-related ICD-10 codes for the first time after January 1, 2004, were tagged as patients with newly diagnosed diabetes. Additionally, only patients with at least 1 additional record of diabetes-related diagnostic codes within one year from the first date of the diabetes diagnosis were included as study participants to improve the accuracy to define a new diabetic patient. Furthermore, we excluded patients who were previously diagnosed with any cancer, stroke, and CVD before diabetes diagnosis. Among those, patients who participated in the national health checkup at least once within 3 years before and after diabetes diagnosis were included in the final study population to assess the status of smoking habit change.

The Institutional Review Board (IRB) of Seoul National University Bundang Hospital approved this study (IRB No. X-1701/378–902) and waived the requirement of informed consents from the study participants due to the anonymity of the data obtained from the NHIS database. All experiments were conducted in accordance with the relevant guidelines and regulations.

### Assessment of status of smoking habit change

The self-reported questionnaire used in the NHIS biennial health checkup contains questions on smoking habits. The status of smoking habit change before and after diabetes diagnosis was determined through comparison of the participant’s answers on the smoking habit questions in the latest survey before the diagnosis to those in the first survey conducted after the diabetes diagnosis. The patients were divided into 6 groups based on the status of smoking habit change and smoking amount: non-reducer, reducer, quitter, never smoker, ex-smoker, and starter. The smoking amount was defined based on the number of cigarettes a participant smokes per day, which was categorized into the following 3 groups: light (1–9 cigarettes/day), moderate (10–19 cigarettes/day), and heavy (≥20 cigarettes/day)^31^. The non-reducer group included smokers who did not reduce their smoking amount even after diabetes diagnosis. In contrast, the reducer group was composed of smokers who reduced their daily smoking amount, for example, from heavy to moderate or light and from moderate to light. Meanwhile, the quitter group consisted of participants who answered that they were current smokers in the health examination survey before the diagnosis but who indicated that they were ex-smokers in the health checkup survey after the diagnosis. The never smoker and ex-smoker groups include participants who answered that they were never smokers and ex-smokers in both pre- and post-health checkups, respectively. Smoking starters referred to patients who answered that they were current smokers in the health checkup survey after diabetes diagnosis but stated that they were never smokers or ex-smokers in the health examination survey before the diagnosis. Thus, the smoking starter group included patients who initiated or relapsed smoking after diabetes diagnosis.

Weight gain after smoking cessation has been widely recognized as one of the reasons why smokers do not attempt to quit smoking^37,38^. Additionally, weight gain is also a sole risk factor of cardiovascular diseases^23^. Thus, to investigate the effects of weight change after smoking cessation along with those of smoking habit change on the risks of all-cause mortality and CVDs among patients with newly diagnosed diabetes, the quitters were further categorized into 3 subgroups based on the status of BMI change after smoking cessation: with BMI loss (≤−1.0 kg/m^2^), without BMI change (−1.0 to <1.0 kg/m^2^), and with BMI gain (≥1.0 kg/m^2^). The cutoff value for the BMI change was determined based on previous studies and the results of preliminary analysis. Previous studies showed varying magnitudes of weight gain after smoking cessation, depending on the study population and designs^21,37,39,40^. Considering that the database used in this study provided only the BMI (not body weight), we calculated the BMI gain, which corresponded to the weight gain after smoking cessation, by using the average height of Korean male aged ≥40 years old. The resulting BMI changes were as follows: 0.7, 1.1, and 1.4 kg/m^2^ for 2, 3, and 4 kg weight gains, respectively. The results of the risk analysis for all-cause mortality with all 3 BMI change cutoff values were consistent (Supplementary Table S2), and the mean BMI gain among patients with newly diagnosed diabetes in this study was 1.0 ± 0.9 kg/m^2^. Hence, the 1.0 kg/m^2^ BMI change was used as the cutoff value.

### Outcomes

The outcomes were all-cause mortality and CVDs after diabetes diagnosis. The all-cause mortality was identified based on the reported date of death. The cardiovascular diseases included strokes and MI. The ICD-10 codes were used in the identification of the incident cases of total strokes (I60–69) and MI (I21–24) from the database. Patients with an event of newly diagnosed strokes or MI were defined as those who were hospitalized for two or more days with at least one of CVD-related codes after diabetes diagnosis to minimize the overestimation of event cases.

### Statistical analysis

The basal characteristics of patients at the pre-health checkup before diabetes diagnosis were expressed as numbers and percentages for categorical variables and median with interquartile range (IQR) for continuous variables. The comparisons between groups were conducted using the Kruskal–Wallis and Pearson’s chi-squared tests for continuous and categorical values, respectively, in the univariate analysis. The crude incidence rates for all-cause mortality, total strokes, and MI were also calculated for each group.

Survival regression analyses, Cox regression and competing-risks survival regression based on Fine and Gray’s model, were conducted to estimate the hazard ratios of all-cause mortality and CVDs respectively. Competing-risk survival regression has been known as a useful alternative to Cox regression in the presence of competing risks. Model 1 for the survival analysis was adjusted only for age, whereas model 2 for the survival analysis was adjusted for age, income status, CCI score, smoking status, alcohol consumption, physical activity, BMI, blood pressure, and fasting serum glucose and cholesterol level at the baseline. The data collection and statistical analyses were conducted using STATA version 14.0 (StataCorp LP, College Station, TX, USA) and Python version 2.7 (Python Software Foundation, Beaverton, OR, USA).

### Data availability

The datasets generated during and/or analyzed during the current study are available from the corresponding author on reasonable request.

## Electronic supplementary material

Supplementary tables
