# Supplementary material for: Effects of smoking habit change on all-cause mortality and cardiovascular diseases among patients with newly diagnosed diabetes in Korea
Source: Sci Rep. 2018 Mar 28;8:5316. doi: 10.1038/s41598-018-23729-0 (PMC5871763; doi:10.1038/s41598-018-23729-0)
Supplement: Supplementary file 1 — Supplementary tables [file 41598_2018_23729_MOESM1_ESM.docx]

**Effects of smoking habit change on all-cause mortality and cardiovascular diseases among patients with newly diagnosed diabetes in Korea**

Mi Hee Cho, Kiheon Lee, Sang Min Park, Jooyoung Chang, Seulggie Choi, Kyuwoong Kim, Hye-Yeon Koo, Ji-Hye Jun, Sung Min Kim

^*^Corresponding author: Kiheon Lee

Table S1. Multivariate-adjusted hazard ratios with 95% confidence intervals of all-cause mortality and cardiovascular disease stratified by age

|  | Non-reducer | | Reducer | | Quitter | | | | | | | | |  |  |  |  |  |  |  |  |
| --- | --- | --- | --- | --- | --- | --- | --- | --- | --- | --- | --- | --- | --- | --- | --- | --- | --- | --- | --- | --- | --- |
|  |  |  |  |  | with BMI loss | | | without BMI change | | | with BMI gain | | |  |  |  |  |  |  |  |  |
|  | aHR^a^ | 95% CI | aHR^a^ | 95% CI | | aHR^a^ | 95% CI | | aHR^a^ | 95% CI | aHR^a^ | 95% CI |  |  |  |  |  |  |  |  |  |
| All-cause mortality |  |  |  |  | |  |  | |  |  |  |  |  |  |  |  |  |  |  |  |  |
| All | 1.00 | - | 1.22 | 0.83-1.79 | | 1.78 | 1.13-2.83 | | 0.68 | 0.46-1.00 | 0.67 | 0.36-1.24 |  |  |  |  |  |  |  |  |  |
| Age |  | | | | | | | | | | | | |  |  |  |  |  |  |  |  |
| <60 | 1.00 | - | 0.91 | 0.50-1.68 | | 0.90 | 0.36-2.24 | | 0.47 | 0.23-0.93 | 0.33 | 0.10-1.04 |  |  |  |  |  |  |  |  |  |
| ≥60 | 1.00 | - | 1.73 | 1.03-2.90 | | 2.48 | 1.45-4.25 | | 0.86 | 0.52-1.43 | 1.18 | 0.55-2.52 |  |  |  |  |  |  |  |  |  |
| Cardiovascular diseases | | | | | | | | | | | | | |  |  |  |  |  |  |  |  |
| All | 1.00 | - | 0.75 | 0.51-1.10 | | 1.09 | 0.71-1.68 | | 0.79 | 0.58-1.07 | 0.94 | 0.60-1.47 |  |  |  |  |  |  |  |  |  |
| Age |  |  |  |  | |  |  | |  |  |  |  |  |  |  |  |  |  |  |  |  |
| <60 | 1.00 | - | 0.51 | 0.28-0.91 | | 0.79 | 0.40-1.58 | | 0.74 | 0.48-1.11 | 0.84 | 0.47-1.46 |  |  |  |  |  |  |  |  |  |
| ≥60 | 1.00 | - | 1.21 | 0.71-2.12 | | 1.58 | 0.88-2.84 | | 0.95 | 0.59-1.54 | 1.22 | 0.57-2.62 |  |  |  |  |  |  |  |  |  |

aHR, adjusted hazard ratio; CI, confidence interval; BMI, body mass index; CCI, Charlson comorbidity index.

^a^ Multivariate-adjusted model was adjusted for age, income status, Charlson comorbidity index score, alcohol consumption, physical activity, smoking status, BMI, blood pressure, and fasting serum glucose and cholesterol levels.

Table S2. Multivariate-adjusted hazard ratios of all-cause mortality by cutoff value of BMI change with newly diagnosed diabetes

|  | Cutoff value of BMI change | | | | | |
| --- | --- | --- | --- | --- | --- | --- |
|  | 0.7 kg/m^2^ | | 1.0 kg/m^2^ | | 1.4 kg/m^2^ | |
|  | aHR^a^ | CI | aHR^a^ | CI | aHR^a^ | CI |
| Non-reducers | 1.00 | - | 1.00 | - | 1.00 | - |
| Reducers | 1.22 | 0.83-1.79 | 1.23 | 0.85-1.79 | 1.22 | 0.83-1.79 |
| Quitters with BMI loss | 1.70 | 1.14-2.54 | 1.58 | 1.03-2.44 | 2.15 | 1.32-3.51 |
| Quitters without BMI change | 0.63 | 0.40-0.98 | 0.63 | 0.43-0.92 | 0.72 | 0.50-1.02 |
| Quitters with BMI gain | 0.69 | 0.34-1.06 | 0.64 | 0.35-1.19 | 0.64 | 0.33-1.31 |

aHR, adjusted hazard ratio; CI, confidence interval; BMI, body mass index

^a^ Multivariate-adjusted model was adjusted for age, income status, Charlson comorbidity index score, alcohol consumption, physical activity, smoking status, BMI, blood pressure, and fasting serum glucose and cholesterol levels.
